# Differential Effects of Vitamins A and D on the Transcriptional Landscape of Human Monocytes during Infection

**DOI:** 10.1038/srep40599

**Published:** 2017-01-17

**Authors:** Tilman E. Klassert, Julia Bräuer, Martin Hölzer, Magdalena Stock, Konstantin Riege, Cristina Zubiría-Barrera, Mario M. Müller, Silke Rummler, Christine Skerka, Manja Marz, Hortense Slevogt

**Affiliations:** 1 Jena University Hospital, Septomics Research Center, Jena, 07745, Germany; 2 Friedrich Schiller University, Bioinformatics/High Throughput Analysis, Jena, 07743, Germany; 3 Center for Sepsis Control and Care (CSCC), Jena University Hospital, Erlanger Allee 101, Jena, 07747, Germany; 4 Jena University Hospital, Institute of Transfusion Medicine, Jena, 07747, Germany; 5 Leibniz Institute for Natural Product Research and Infection Biology, Hans Knöll Institute, Department of Infection Biology, Jena, 07745, Germany

## Abstract

Vitamin A and vitamin D are essential nutrients with a wide range of pleiotropic effects in humans. Beyond their well-documented roles in cellular differentiation, embryogenesis, tissue maintenance and bone/calcium homeostasis, both vitamins have attracted considerable attention due to their association with-immunological traits. Nevertheless, our knowledge of their immunomodulatory potential during infection is restricted to single gene-centric studies, which do not reflect the complexity of immune processes. In the present study, we performed a comprehensive RNA-seq-based approach to define the whole immunomodulatory role of vitamins A and D during infection. Using human monocytes as host cells, we characterized the differential role of both vitamins upon infection with three different pathogens: *Aspergillus fumigatus, Candida albicans* and *Escherichia coli*. Both vitamins showed an unexpected ability to counteract the pathogen-induced transcriptional responses. Upon infection, we identified 346 and 176 immune-relevant genes that were regulated by atRA and vitD, respectively. This immunomodulatory activity was dependent on the inflammatory stimulus, allowing us to distinguish regulatory patterns which were specific for each stimulatory setting. Moreover, we explored possible direct and indirect mechanisms of vitamin-mediated regulation of the immune response. Our findings highlight the importance of vitamin-monitoring in critically ill patients. Moreover, our results underpin the potential of atRA and vitD as therapeutic options for anti-inflammatory treatment.

Vitamin A and vitamin D are known to exert pleiotropic effects on many biological processes^1^,^2^. Their broad functional potential relies mainly on their capacity to facilitate transcriptional changes by binding their specific nuclear receptors: the retinoic acid receptors (RARs) and the vitamin D receptor (VDR), respectively^1^,^2^,^3^,^4^,^5^. While vitamin A is involved in functions such as reproduction, embryogenesis, cellular differentiation, and maintenance of body tissues^6^,^7^,^8^, vitamin D is crucial for the regulation of bone and calcium homeostasis^9^. Beside these well-known functions, both vitamins have been associated with a regulatory role in immunity^10^,^11^. Over the last decades, an increasing effort has been devoted to better define the involvement of both vitamins in the regulation of the immune response. Especially, since vitamin A deficiency and seasonal variations in vitamin D levels have been associated with an increased susceptibility to severe infectious diseases^12^,^13^,^14^.

Animal models and clinical trials have demonstrated the protective effect of vitamin supplementation in several infectious diseases^15^,^16^,^17^,^18^. Indeed, vitamin A or vitamin D supplementation has been proposed as treatment option in diseases such as measles and tuberculosis, respectively^15^,^19^. Meanwhile, *in vitro* studies have attempted to characterize the mechanisms underlying such protective effects. Most studies pointed towards an immunomodulatory effect of both vitamins, after the identification of candidate genes regulated by vitamin A or vitamin D metabolites^10^,^11^. In this sense, vitamin A has been shown to decrease LPS-induced expression of pro-inflammatory cytokines such as TNF*α* and IL-6, or chemokines like MIP-1*α* and MIP-1*β* in human macrophages and dendritic cells^20^. In our group, we could recently demonstrate a similar effect in monocytes upon fungal infection, reporting the down-regulation of *Candida albicans*-induced TNF*α*, IL-6 and IL-12B expression in response to all-trans retinoic acid (atRA), the active metabolite of vitamin A^21^. Meanwhile, most of the current understanding of vitamin D-mediated immunomodulation derives from studies related to *Mycobacterium tuberculosis* infections^22^. Beside its ability to induce the expression of anti-microbial peptide Cathelicidin (CAMP) or *β*-defensins^11^,^22^, vitamin D is also able to regulate the expression of TNF*α* and IL-6 in human monocytes upon bacterial stimulation^23^. In fungal infections, vitamin D has also been shown to modulate the production of cytokines such as IL-6, TNF*α*, IL-17, and IFN*γ* in monocytes^24^.

Although these studies are evidence for the significant impact of vitamins on the immune response of human leukocytes to bacterial and fungal pathogens, they are all qPCR-based (i.e. single gene-centric) and therefore limited. Moreover, some findings of candidate genes for the vitamin-mediated control of immune functions could not be replicated between studies. For example, Oeth *et al*.^25^ could not detect any atRA-mediated changes in the TNF*α* production by monocytes after stimulation with LPS^25^. In addition, single gene-centric studies cannot exhibit the complexity of cellular interactions and pathways regulating immune processes.

In the present work, we performed a high-throughput approach based on RNA sequencing to define the whole immunomodulatory potential of vitamins A and D during infection. Therefore, we analysed their differential impact on infections of bacterial and fungal origin. The bacterium *Escherichia coli* is one of the most common etiologic agents of sepsis^26^, while *Candida albicans* and *Aspergillus fumigatus* are among the most important causes of systemic mycoses^27^. During these systemic infections, monocytes play a central role in the host defense contributing not only to pathogen recognition, but also as phagocytes and effector cells^28^. Hence, in the present and exhaustive study, we analysed the noteworthy immunomodulatory role of vitamins on human monocytes.

## Methods

AtRA and 1*α*,25(OH)2D3 (vitamin D) were purchased from Sigma-Aldrich (Germany) and dissolved in absolute ethanol. FITC-conjugated monoclonal mouse anti-human CD14 antibody, APC-conjugated monoclonal mouse anti-human CD16 antibody, and FITC-conjugated mouse IgG1 *κ* isotype control antibody were purchased from eBioscience (USA). APC-conjugated mouse IgG1 *κ* isotype control antibody was purchased from Biolegend (USA).

### Preparation of fungi and bacteria

Overnight cultures of *C. albicans* (SC5314) in YPD medium were washed three times with PBS and resuspended at 10^8^ yeasts/ml in RPMI 1640 GlutaMAX medium (Gibco, UK) supplemented with 10% fetal bovine serum (FBS; Biochrom, Germany).

*A. fumigatus* (AF293) was grown on AMM plates at 30 °C for 6 d. Conidiospores were harvested by rinsing the plates with water +0.05% Tween-20 (Sigma-Aldrich, Germany) and filtered through 70-*μ*m and 30-*μ*m pre-separation filters (Miltenyi Biotec, UK) to obtain a single-cell suspension. Conidia were then washed twice in PBS and resuspended at 10^7^ conidia/ml in RPMI 1640 GlutaMAX medium supplemented with 10% FBS (Biochrom, Germany). Germlings were obtained by incubation of conidia at 37 °C under continuous shaking for 6–8 h. They were then centrifuged and resuspended at 10^8^ cells/ml in fresh RPMI 1640 GlutaMAX medium supplemented with 10% FBS.

Overnight culture of *Escherichia coli* (isolate 018:K1:H7) in LB medium was washed three times in PBS and resuspended in RPMI 1640 GlutaMAX medium supplemented with 10% FBS. The concentration of bacteria was adjusted to 10^9^ cfu/ml. All pathogens were heat-killed by incubation at 65 °C for 30 min and immediately used for stimulation assays.

### Monocyte isolation

Human monocytes were isolated from 500 ml fresh whole blood (drawn within 1 h before use) of healthy male donors. Blood was layered onto an equal volume of 1-Step Polymorphs (Accurate Chemical & Scientific Corporation, USA) and centrifuged at 650 × *g* for 35 min. After centrifugation, the peripheral blood mononuclear cells (PBMCs) were collected, and normal osmolarity was restored by adding an equal volume of 0.45% cold NaCl. After erythrocyte lysis using a hypotonic buffer, cells were washed twice in cold PBS and counted using a Neubauer chamber. Cell viability of >95% was assessed by trypan blue staining. Monocytes were isolated from the PBMCs using the monocyte isolation kit II and quadro-MACS (Miltenyi Biotec, UK), following manufacturer’s instructions.

### Ethics statement

The blood of healthy male donors was drawn after written informed consent. This is in accordance with the Declaration of Helsinki, all protocols were approved by the Ethics Committee of the University Hospital Jena (permit number: 3639-12/12).

### Flow cytometry

**Figure 1:**
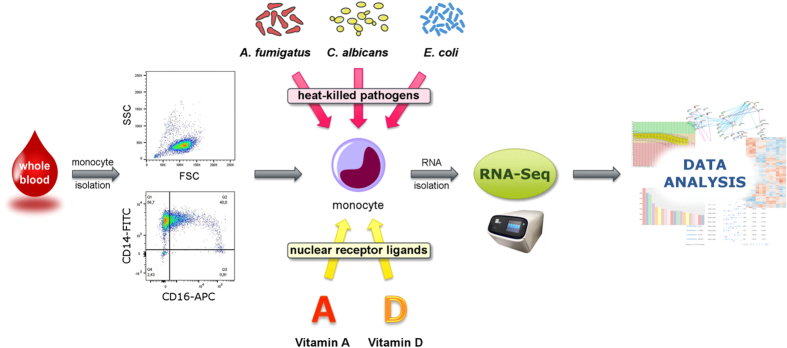
Experimental workflow. Human monocytes were isolated from fresh whole blood and purity of the cells was analyzed by flow cytometry. Upper scatterplot: Forward scatter (FSC) and side scatter (SSC) measurement. Lower scatterplot: Fluorescence intensities of cells stained with FITC-conjugated CD14 antibody and APC-conjugated CD16 antibody. Monocytes were then pre-incubated with vitamin A (atRA) or vitamin D, followed by stimulation with heat-killed *A. fumigatus, C. albicans* or *E. coli* for 6 h. Poly-(A) RNA was isolated from the monocytes and subjected to RNA sequencing.

To analyse the purity of the isolated human monocytes, these were washed with PBS containing 10% FBS and stained with FITC-conjugated mouse anti-human CD14 antibody (1 *μ*g/100 *μ*l, 30 min) and APC-conjugated mouse anti-human CD16 antibody (0.03 *μ*g/100 *μ*l, 30 min). The corresponding fluorophore-conjugated mouse IgG1 *κ* antibodies were used as isotype control. Samples were measured on a FACSAria II apparatus (BD Biosciences, USA) and data were analysed using the FLOWJO 7.6.4 software. The resulting mean fluorescence intensities (MFIs) were normalized to those of unstained cells in each case. The purity of the obtained monocytes was >90% (see Fig. 1).

### Stimulation assays

Monocytes were resuspended at 5 × 10^6^ cells/ml in RPMI 1640 GlutaMAX medium (Gibco, UK) supplemented with 10% FBS (Biochrom, Germany) and 1% Penicillin/Streptomycin (Thermo Fisher Scientific, USA). They were seeded on 6-well plates (VWR International, Germany) and allowed to equilibrate at 37 °C and 5% CO_2_ for 2 h. Cells were then pre-incubated with 1 *μ*M atRA or 1*α*,25(OH)_2_D_3_ for 30 min. Then, the heat-killed pathogens were added at a pathogen:host ratio of 1:1 for *C. albicans* yeast and *A. fumigatus* germ tubes, and 10:1 in case of *E. coli* stimulation. After 6 h of incubation at 37 °C and 5% CO_2_, cell viability >90% was assessed by trypan blue staining, and the monocytes were harvested for RNA isolation. The whole experimental workflow is depicted in Fig. 1.

In total, we had four different immune-stimulatory settings (w/o infection, *A. fumigatus* infection, *C. albicans* infection and *E. coli* infection), in each of which we aimed to address the effect of vitamin A (atRA) or vitamin D supplementation.

### RNA sequencing

RNA was isolated from 5 × 10^6^ monocytes using the RNeasy Mini Kit (Qiagen, Germany). An additional step was included to remove the residual genomic DNA using DNaseI (Qiagen, Germany). Total RNA was quantified using a Nanodrop ND-1000 spectrophotometer (Thermo Fisher Scientific, USA). The quality of the RNA samples (RNA Integrity Number (RIN) values ≥ 7.0) was measured using a Tape Station 2200 (Agilent Technologies, USA). Poly-(A) RNA was purified from 2 *μ*g of total RNA using the Dynabeads mRNA DIRECT Micro Purification Kit (Thermo Fisher Scientific, USA), according to manufacturer’s instructions. Quality control for the depletion of rRNA was carried out using High Sensitivity RNA Screen Tapes (Agilent Technologies, USA).

Strand-specific whole transcriptome libraries were prepared using the Ion Total RNA-Seq Kit v2.0 (Thermo Fisher Scientific, USA). RNAse III was employed to fragment the purified RNA. Ion adapters were ligated to the resulting fragments, and reverse transcription was performed using the SuperScript III Enzyme Mix (Thermo Fisher Scientific, USA). Barcoded primers were used to amplify the libraries with the Platinum PCR High Fidelity polymerase (Thermo Fisher Scientific, USA). Size distribution analysis and quantification of the final barcoded libraries was performed on D1000 Screen Tapes on the Tape Station 2200 (Agilent Technologies, USA). Library templates were clonally amplified on Ion Sphere particles using the Ion PI Hi-Q Chef Kit and Ion Chef instrument (Thermo Fisher Scientific, USA), loaded onto Ion PI Chips and sequenced on an Ion Proton Sequencer (Thermo Fisher Scientific, USA). For sequencing, in total 48 samples were multiplexed on 12 chips. The raw sequence data in fastq format are stored in the Sequence Read Archive (SRA) at National Center for Biotechnology Information (NCBI) and can be accessed at NCBI homepage (https://www.ncbi.nlm.nih.gov/; accession number: SRP076532).

### Bioinformatic analysis of RNA-seq data

#### Quality check and trimming of raw sequence data

The raw data in fastq format were checked with regard to quality (FastQC)^29^ and, thereafter, trimmed with a window size of 10 using Prinseq^30^. After removing rRNA reads, the trimmed reads were mapped onto the human genome version GRCh38 with Segemehl^31^ as described in Riege *et al*.^32^ with default parameters and the –splits option. We used HTSeq-Count^33^ (v0.6.0) to quantify strand-specific and uniquely mapped reads at the exon level. As reference, the full human Ensembl annotation (version GRCh38.80) was chosen, including both protein- and non-coding genes. Thereafter, the mapped reads were counted per gene and per sample at the exon level and used as input for statistical analyses carried out with DESeq2^34^ and various Bioconductor^35^ packages in R.

In order to filter out low-expressed mRNAs, we calculated transcripts per kilobase million (TPM) to eliminate potential biases due to transcript length in normalized read counts^36^. Subsequently, for each stimulatory setting we used TPM = 5 as a minimum limit for detectability^37^ of transcripts. For more detailed information on read processing, see publication by Riege *et al*.^32^.

#### Differential gene expression and key pathway analysis

Pairwise comparisons were carried out to address the effect of each pathogen stimulation (unstimulated samples *versus* pathogen-stimulated samples) and also the effect of the vitamin-mediated regulation in each stimulatory setting (pathogen-stimulated samples *versus* pathogen-/vitamin-stimulated samples).

K-means clustering was performed on variance-stabilized read counts to build a heatmap (selected gene set with adjusted p-value ≤0.05) in R. For this, the pheatmap function was applied with the kmeans option and euclidean clustering distance of the rows. Beforehand, the model-based optimal number of clusters was determined using Mclust of the mclust package in R^38^,^39^. The assigned genes of the resulting clusters were annotated by gene ontology analysis using the PANTHER (Protein ANalysis THrough Evolutionary Relationships) classification system (http://pantherdb.org/; http://geneontology.org/40,41) and the Partek Genomics Suite 6.6 (Partek, USA). Pathway analysis was performed using the Partek Pathways software tool (Partek, USA) which employs the Kegg pathway database. Furthermore, for all genes that are affected by either atRA or vitamin D treatment during any infection setting, STRING networks were generated using the STRING database^42^ (http://string-db.org). In order to identify key pathways involved in either atRA- or vitD-mediated immunomodulation across the three infections, we have applied the KeyPathwayMiner tool^43^,^44^. For each vitamin, we used the log_2_ fold changes derived from the comparisons of pathogen-stimulated samples *versus* pathogen-/vitamin-stimulated samples. All three input tables for each pathogen (and each vitamin, respectively) were logically connected with AND, and the parameters K and L were kept as default. All nodes of the resulting networks represent either genes significantly down-regulated by the vitamins, or were inferred by KeyPathwayMiner to connect subnetworks.

#### In silico analysis of response elements

We performed genome-wide *in silico* analysis of consensus retinoic acid response element (RARE) and vitamin D response element (VDRE) motifs in the proximity of protein-coding genes using RNAbob^45^. The screening window was limited to the regions ±10 kb from transcription start sites and gene ends, according to previous studies^46^. As target RARE motif, we used the recurrent DR5 motif RGKTSAnnnnnRGKTSA, and for VDRE screening the consensus motif RRGKTCAnnRRGKTCA (in IUPAC code). The enrichment of RAREs and VDREs in the proximity of atRA- or vitD-differentially regulated genes was determined using Fisher’s exact test, comparing hits in differentially expressed genes (DEGs) to those in non-DEGs.

### Reverse transcription and quantitative PCR

Stimulation assays were repeated for an earlier time point. After three hours of stimulation, RNA was isolated as previously described. Complementary DNA (cDNA) was synthesized from 1.5 *μ*g of RNA using the High Capacity cDNA Reverse Transcription Kit (Applied Biosystems, UK) following manufacturer’s instructions. For PCR analysis, specific primers for each target gene were designed using the online Primer-BLAST tool of the National Center for Biotechnology Information (NCBI, http://www.ncbi.nlm.nih.gov/tools/primer-blast/). In order to improve the PCR efficiency, possible secondary structures of the amplicons were taken into account by characterizing their nucleotide sequence using the Mfold algorithm^47^. The sequences of all primers used for amplification are listed in Supplementary Table S1.

To quantify the relative expression of each gene, a Corbett Rotor-Gene 6000 (Qiagen, Germany) was used as RealTime qPCR apparatus. Each sample was analysed in a total reaction volume of 20 *μ*l containing 10 *μ*l of 2× SensiMix SYBR Master Mix (Bioline, UK) and 0.2 *μ*M of each primer. All qPCRs were set up using a CAS-1200 pipetting robot (Qiagen, Germany). The cycling conditions were 95 °C for 10 min followed by 40 cycles of 95 °C for 15 s, 60 °C for 20 s and 72 °C for 20 s. For each experiment, an RT-negative sample was included as control. The specificity of the qPCRs was assessed by melting curve analysis. The relative expression of the target genes was analysed using a modified Pfaffl method^48^,^49^. To determine significant differences in the mRNA expression between different experimental conditions, the relative quantity (RQ) for each sample was calculated using the formula 1/E^Ct^, where E is the efficiency and Ct the threshold cycle. The RQ was then normalized to the housekeeping gene peptidylprolyl isomerase B (PPIB). The stability of the housekeeping gene was assessed using the BestKeeper algorithm^50^. The normalized RQ (NRQ) values were log_2_-transformed for further statistical analysis with GraphPad PRISM v5.0. Statistical analysis was performed using repeated measures ANOVA and Bonferroni correction.

## Results

By using an RNA-seq-based approach, we assessed the impact of different fungal and bacterial pathogens on the transcriptional response of human monocytes, with a focus on the potential of vitamins A and D to modulate this response (workflow depicted in Fig. 1). This modulatory role was explored after six hours of stimulation in four different settings: without infection, upon *A. fumigatus* stimulation, upon *C. albicans* stimulation, and upon *E. coli* stimulation.

**Figure 2:**
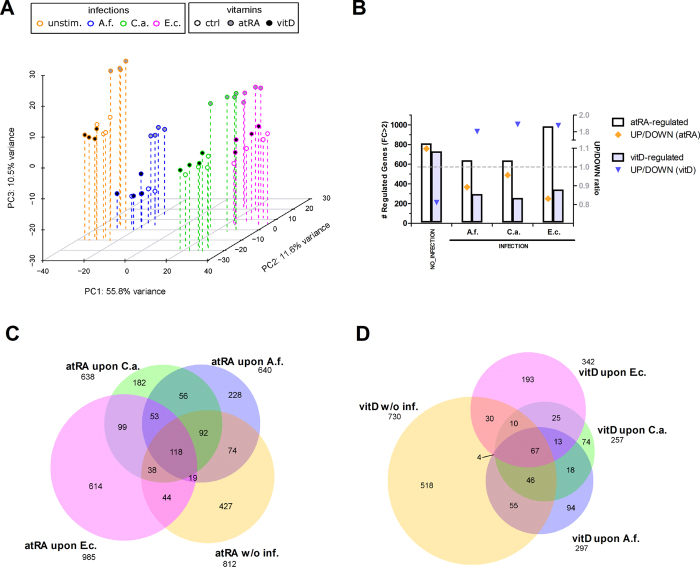
Bird’s eye view of transcriptome changes upon stimulation with vitamin during infection. Our results demonstrate a huge impact of the pathogens and vitamins on the transcriptional landscape of human monocytes. Furthermore, the transcriptional regulation by the vitamins is dependent on the pathogenic stimulus. (**A**) 3-dimensional Principal Component Analysis (3D-PCA) of the top 300 most variant genes was plotted with the scatterplot3d package in R^67^. The first three principal components (PC1-PC3) account for 78% of the total variance of the data. (**B**) Representation of the total number of genes as bars (left Y-axis) and the ratio of up-/down-regulated genes as diamonds and triangles (right Y-axis) in response to atRA and vitD during all stimulatory settings. (**C**) Venn diagram showing the overlap of the atRA-regulated genes (*FC* > 2, *p* < 0.05) during *A. fumigatus* stimulation (A.f., blue), *C. albicans* stimulation (C.a., green), *E. coli* stimulation (E.c., magenta) or in absence of pathogen stimulation (w/o inf., orange). (**D**) Venn diagram showing the overlap of the vitD-regulated genes (*FC* > 2, *p* < 0.05) during *A. fumigatus* stimulation (A.f., blue), *C. albicans* stimulation (C.a., green), *E. coli* stimulation (E.c., magenta) or in absence of pathogen stimulation (w/o inf., orange).

In the entire transcriptional dataset, a total of 6076 protein-coding genes showed significant differential expression (*p* < 0.05) in any of the comparisons conducted. Principal component analysis (PCA) disclosed the immunological challenge as the main source of variance observed in our dataset, as demonstrated by the strong regulatory impact of the pathogens (PC1, Fig. 2). Nevertheless, we could also observe a significant effect of the vitamins on the transcriptional regulation, especially for vitamin A (atRA) (PC2 and PC3; Fig. 2).

### Transcriptional regulation by vitamins is altered during infection

Next, we assessed the total amount of genes regulated by vitamins (*FC* > 2; *P* < 0.05) in each of the immune-stimulatory settings (no infection, *A. fumigatus* infection, *C. albicans* infection and *E. coli* infection), as well as the direction of the regulation (up- *vs.* down-). While vitamin A stimulation led to a predominant up-regulation of gene expression in absence of infection, vitamin D was more active at repressing gene expression (Fig. 2B). Upon immunological challenge, this situation was reversed, with atRA showing higher potential as repressor of gene expression. In contrast, the number of vitD-regulated genes decreased drastically upon infection, and the up-regulated genes almost doubled the down-regulated ones (Fig. 2B). These results suggest that the impact of vitamins on the transcriptional profile of monocytes might be highly dependent on whether the cells are under immune challenge or not. This hypothesis is reinforced when we compare the genes regulated by the vitamins in each of the immune-stimulatory settings (Fig. 2C,D). For vitamin A, we observed that 118 genes were regulated by atRA in all different settings. Nevertheless, we also observed a surprisingly high number of genes specifically regulated in each of the settings, with up to 614 genes regulated by atRA exclusively upon *E. coli* infection, 182 upon *C. albicans* challenge and 228 upon *A. fumigatus* infection (Fig. 2C). In the case of vitamin D, it is notorious that the regulatory potential is reduced upon infection. A total of 518 genes were exclusively regulated by vitD in the absence of immune challenge. Moreover, only 67 (5.8%) of the vitD-regulated genes were shared by all different settings, again suggesting a high dependency on the stimulatory environment.

### Immunomodulation as primary function of vitamins upon infection

**Figure 3:**
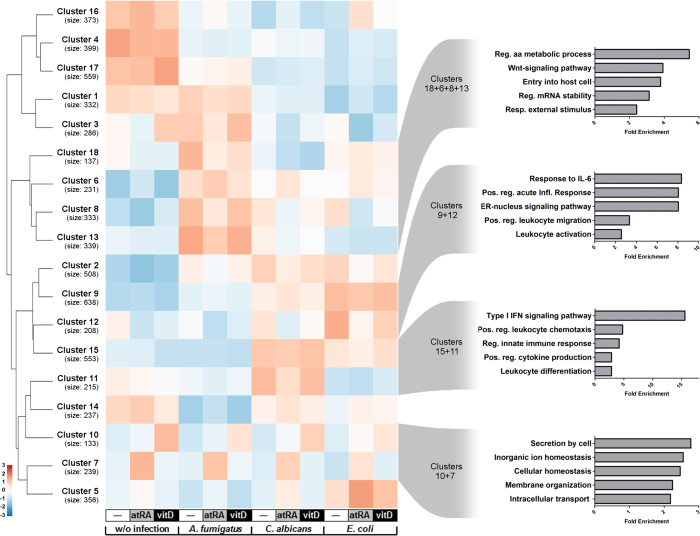
Heatmap of K-means clustering of DEGs and subsequent GO enrichment analysis. K-means clustering was performed on variance-stabilized read counts to build a heatmap for the 6076 differentially expressed protein-coding genes (adjusted p-value < 0.05). *A priori*, the model-based optimal number of *K* = 18 was determined. The clustering of the rows is based on euclidean distance. The colors in the map represent row-scaled expression levels: blue indicates the lowest expression, white indicates intermediate expression, and red indicates the highest expression. Selected clusters were analysed with regard to their biological function by GO enrichment analysis. Most enriched GO categories are shown for representative groups of clusters displaying their fold enrichment.

K-means clustering of the 6076 differentially expressed genes (DEGs) coupled with gene ontology (GO) enrichment analysis allowed us to gain insight into the functional relevance of different groups of vitamin-regulated genes, depending on their role during infection (Fig. 3; Supplementary Dataset S1).

Clusters with a strong stimulatory effect of vitamins A or D, but little impact of the immune challenge, were governed by genes involved in cellular processes, such as inorganic ion homeostasis, membrane organization or intracellular transport (Cluster 7 and 10; Fig. 3). Interestingly, the regulatory impact of the vitamins on these clusters seems to regress as infection comes into play, especially during bacterial challenge. On the other hand, and as expected, gene clusters displaying a strong impact of the different pathogens were composed of genes belonging to immune-relevant GO categories. Clusters that were characterized by a main effect of *A. fumigatus* challenge (Cluster 6, 8, 13 and 18; Fig. 3) were governed by genes involved in amino acid metabolism, the immune-relevant Wnt-pathway signaling and mechanisms of entrance into the host cell, among others. Clusters 11 and 15 (Fig. 3) are composed by genes mainly related to the defense against *C. albicans*. In these clusters we could identify a highly significant overrepresentation of genes belonging to the type-I IFN signaling pathway, leukocyte chemotaxis and cytokine production. A main impact of *E. coli* stimulation was observed in clusters 9 and 12 (Fig. 3), characterized by genes relevant in the IL-6 response, leukocyte migration and leukocyte activation, among others. Importantly, in most of these gene clusters we could identify an important regulatory contribution by both vitamins, especially for vitamin A. Moreover, in several clusters the impact of both vitamins became apparent only upon pathogen stimulation, suggesting a differential role of vitamins during infection when compared to cellular homeostasis (i.e. in absence of immunological challenge).

**Figure 4:**
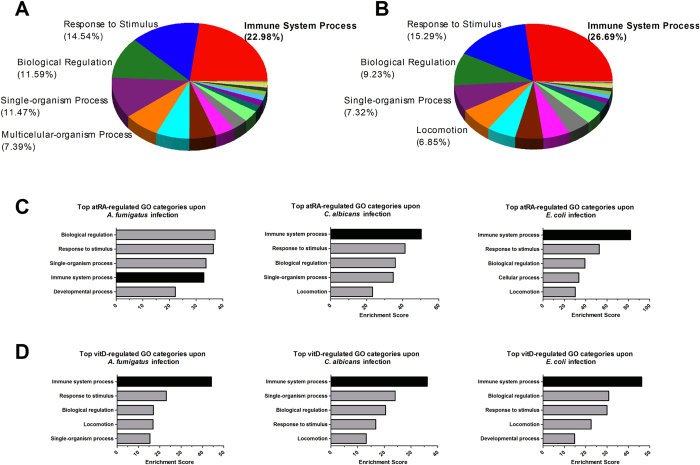
Gene ontology analysis of the vitamin-induced transcriptional changes during infection. Analysis revealed the Immune System Process as the most affected GO category in response to vitamin treatment. (**A**) GO enrichment analysis of all atRA-regulated genes (1573 genes, *FC* > 2, *p* < 0.05) during any of the three analysed infections. Percentage of the total enrichment scores are shown for the top five GO categories (biological process). (**B**) GO enrichment analysis of all vitD-regulated genes (624 genes, *FC* > 2, *p* < 0.05) during any of the three analysed infections. (**C**) Top atRA-regulated GO categories during each of the infections. (**D**) Top vitD-regulated GO categories during each of the infections.

Subsequently, we performed GO analysis on all vitamin A- or vitamin D-regulated genes (*FC* > 2; *p* < 0.05) during infection. A total of 1573 genes were differentially regulated by atRA under any of the pathogen settings. GO analysis of these genes revealed the Immune System Process (GO:0002376) as the most enriched GO category, followed by Response to Stimulus (GO:0050896) (Fig. 4A). A similar enrichment was obtained also for the 624 vitD-regulated genes (Fig. 4B), demonstrating the important role of both vitamins in immune processes. Moreover, Immune System Process was also the most prominent category among the genes regulated by atRA during *C. albicans* and *E. coli* infections when settings were analysed separately (Fig. 4C). For vitamin D, Immune System Process was the top-enriched GO category upon all settings. In addition, most of the immune-relevant genes regulated by vitamins A and D are highly expressed in monocytes (Supplementary Fig. S1). Kegg-pathway analysis of vitamin-regulated genes showed the Cytokine-Cytokine Receptor Interaction as the pathway with highest enrichment score for both vitamins (Supplementary Table S2). Other significantly enriched pathways included Chemokine signaling, TNF signaling and Hematopoietic cell lineage, among several other immune-relevant processes. Thus, pathway analysis underpins the remarkable impact of the vitamins on immune functions.

### Counteracting the transcriptional response against pathogens

**Figure 5:**
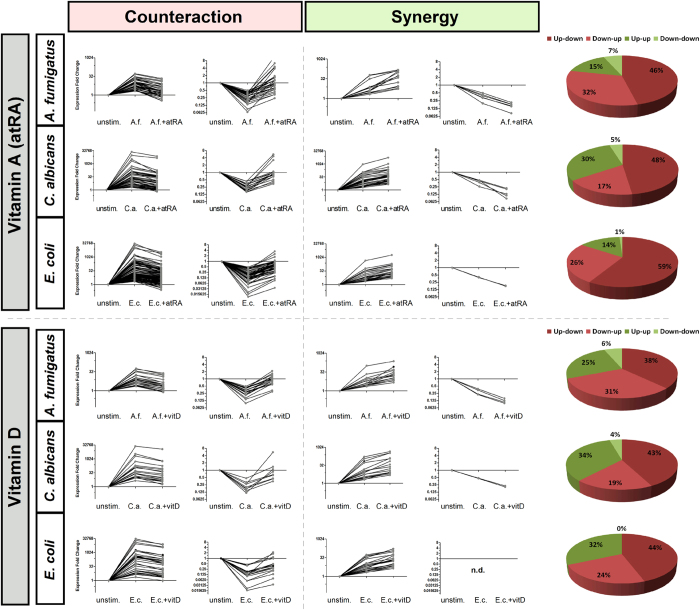
Vitamins A and D strongly counteract the transcriptional response of human monocytes to pathogens. Graphic representation of the expression dynamic of immune-relevant genes (GO:0002376) differentially regulated by both the pathogens and the vitamins. Patterns are divided by the type of correlation observed between the effects of pathogen and vitamin stimulations: counteractive effect (up-regulation by pathogen and down-regulation by vitamin, or *vice versa*) and synergistic effect (same direction observed in the differential expression induced by pathogen and vitamin stimulations). Pie charts show the proportion of genes depicting counteractive effects (red) and synergistic effects (green).

The question remained as to the direction and extent of the vitamin-mediated regulation in each stimulatory setting. In order to address these questions, we subsequently analysed the differential expression of all those immune-relevant genes that were regulated by both the vitamins and the pathogens. Interestingly, there was a huge overlap of genes regulated by both stimuli. Thus, of the 235 immune-relevant genes (GO:0002376) that were regulated by atRA during *E. coli* infection, up to 195 genes (83%) were also regulated by the pathogen itself. Similar overlaps were observed during fungal infections with up to 70.5% and 72.7% for *A. fumigatus* and *C. albicans* stimulation, respectively. For vitamin D, these overlaps with the infections were 73.6%, 64.4% and 74.2% for *A. fumigatus, C. albicans* and *E. coli* challenge, respectively. By plotting fold changes relative to their unstimulated controls, we could discriminate between counteractive and synergistic effects between the vitamins and the pathogenic stimulus in each case (Fig. 5).

In all settings, the vast majority of the immune-relevant genes were up-regulated after pathogen challenge, as expected, and this effect was reversed by the vitamins. Especially atRA showed an important counteractive effect against the pathogen challenge. AtRA counteracted the effect of the pathogens in 78% of the genes regulated also by *A. fumigatus*, 65% of the genes regulated by *C. albicans*, and 85% of the genes regulated by *E. coli*. Similar results were obtained for vitD-mediated regulation, with 69%, 62% and 68%, respectively (Fig. 5). Moreover, this effect becomes even more apparent when analysing the expression of genes belonging to the GO category Immune Response (GO:0006955), especially in the case of vitamin A (see Supplementary Fig. S2). This significant counteractive effect suggests an important immunomodulatory potential for both vitamins during bacterial and fungal infections.

### Differential role of vitamins A and D as immunomodulators

#### Vitamin-dependent regulation of inflammatory mediators during infection

**Figure 6:**
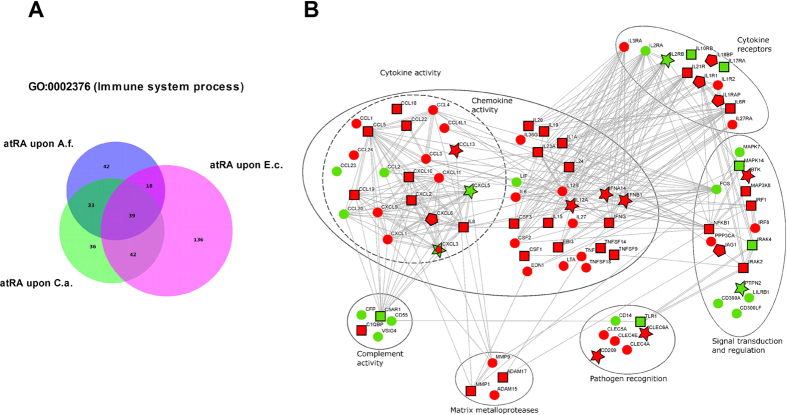
Immunomodulatory footprint of vitamin A during infection. AtRA shows a pathogen-specific regulatory role on immune-relevant genes leading to an overall down-regulation of cytokines, chemokines and matrix metalloproteases and an up-regulation of complement-related genes. (**A**) Venn diagram showing the overlap and amount of atRA-regulated immune-relevant genes (GO:0002376) in each of the infections analysed: *A. fumigatus* (blue), *C. albicans* (green) and *E. coli* (magenta). (**B**) Network based on experimental and database-derived knowledge (edges) generated with the STRING database. ○ atRA regulated these genes in at least two of the pathogenic infection settings; 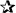 atRA-regulated genes during *C. albicans* infection; □ atRA-regulated genes during *E. coli* infection; 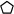 atRA-regulated genes during *A. fumigatus* infection; red: down-regulation, green: up-regulation.

Across all three pathogen settings, atRA was able to significantly modulate (*FC* > 2; *p* < 0.05) the expression of 346 genes belonging to GO:0002376 (Immune System Process). Of these genes, 39 were common for all three infections, whereas 42 genes were regulated by atRA only upon *A. fumigatus* infection and 36 during *C. albicans* infection. Upon *E. coli* challenge, atRA regulated significantly more genes, with 136 being specific for that infection (Fig. 6A). These genes included cytokines such as IL1A, IL15, IL19, IL20, IL23A, IL24, CSF1 and IFNG, chemokines like CXCL10 and IL8, as well as metalloproteases such as MMP1, among others (Fig. 6B, Supplementary Dataset S2). Genes exclusively regulated by atRA during fungal infections included CXCL6 for *A. fumigatus* challenge, and the fungal pattern recognition receptor Dectin-2 (CLEC6A) as well as the type-I interferons IFNB1 and IFNA14 during *C. albicans* infection. Also the cytokine-coding gene IL12A was regulated by atRA exclusively upon *C. albicans* challenge, ranking among the top down-regulated genes in the whole dataset (*FC* = −42.2, adjusted *p* = 1.9*E* − 049; Supplementary Dataset S2).

As shown in Fig. 6B, atRA led to an important down-regulation of several immune-relevant genes when compared to their expression upon pathogen challenge alone. Almost all cytokines were down-regulated by atRA, and a similar pattern was observed for the chemokines and metalloproteases. Interestingly, genes involved in complement activity, such as C5AR1, were rather up-regulated by addition of atRA. Among the top up-regulated genes we also identified a member of the immunoregulatory CD300 molecules, CD300A, up-regulated by atRA in all settings, up to 32-fold (see Supplementary Dataset S2).

**Figure 7:**
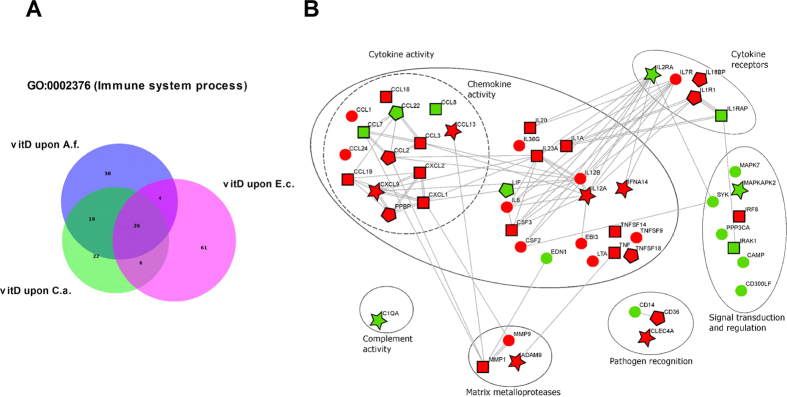
Immunomodulatory footprint of vitamin D during infection. VitD shows a pathogen-specific regulatory role on immune-relevant genes leading to an overall down-regulation of cytokines, chemokines and matrix metalloproteases. (**A**) Venn diagram showing the overlap and amount of vitD-regulated immune-relevant genes (GO:0002376) in each of the infections analysed: *A. fumigatus* (blue), *C. albicans* (green) and *E. coli* (magenta). (**B**) Network based on experimental and database-derived knowledge (edges) generated with the STRING database. ○ vitD regulated these genes in at least two of the pathogenic infection settings; 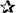 vitD-regulated genes during *C. albicans* infection; □ vitD-regulated genes during *E. coli* infection; 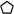 vitD-regulated genes during *A. fumigatus* infection; red: down-regulation, green: up-regulation.

Vitamin D regulated less immune-relevant genes (GO:0002376) than atRA, with a total of 176 genes across all three settings. 38 were specific upon *A. fumigatus* infection, 22 upon *C. albicans* infection and 61 upon *E. coli* challenge. 26 of the vitD-regulated genes were common to all stimulatory settings (Fig. 7A). As shown in Fig. 7B, we can also observe a rather down-regulatory effect of vitD on cytokines, chemokines and metalloproteases. Several of the cytokines regulated by atRA were also regulated in the same way by vitD. These included IL6, IL1A, IL12B, CCL1, CXCL1 and CXCL2, among others. Other immune-relevant genes were exclusive for vitD regulation, such as the antimicrobial peptide Cathelicidin (CAMP) or the chemokine CCL8 (Fig. 7B; Supplementary Dataset S2), both up-regulated by vitD.

Taking all together, both vitamins show an important role as modulators of the transcriptional response against fungi and gram-negative bacteria, with a strong impact on specific cytokine- and chemokine-expression, depending on the stimulatory setting. Moreover, for both vitamins, we could identify consensus inhibitory networks across the three different pathogenic stimulations using the KeyPathwayMiner^43^,^44^ tool. These networks confirmed the important regulatory role of both vitamins on the TNF signaling, metalloprotease production, and IFN pathways (see Supplementary Fig. S3).

To address whether transcriptional mechanisms might indirectly contribute to the vitamin-mediated immunomodulation, we investigated the expression profiles of central regulators of relevant signaling cascades at an earlier stage. We were able to identify several genes which were differentially expressed in response to atRA after three hours of stimulation. These included the activating kinase BTK and the phosphatase PPP3CA, as well as the inhibitory phosphatases PTPN7, DUSP1 and DUSP7. Also the regulatory receptors LILRB1 and CD300A were already regulated by atRA at this early stage (see Supplementary Fig. S4).

#### Comparison of the immunomodulatory potential of atRA and vitD

**Figure 8:**
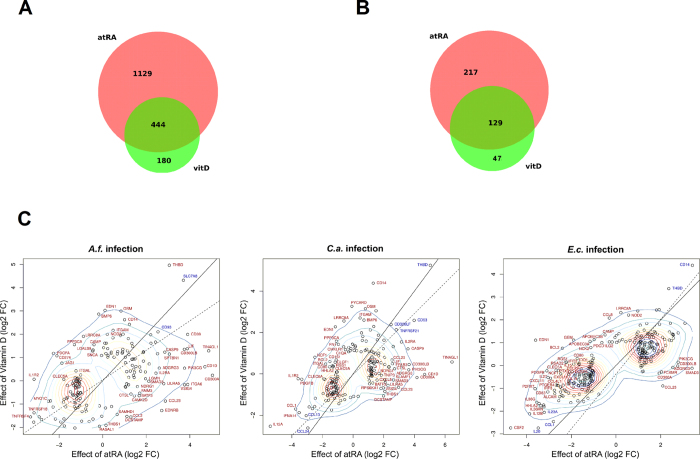
Comparison of the immunomodulatory potentials of vitamin A and vitamin D. During infection, an important overlap among the gene sets regulated by both vitamins could be revealed. Nevertheless, differential modulatory effects by each of the vitamins could be addressed among several immune-relevant genes. (**A**) Venn diagram showing the overlap of atRA- and vitD-regulated protein-coding genes during any of the infections. (**B**) Venn diagram showing the overlap of atRA- and vitD-regulated genes belonging to GO category GO:0002376 (Immune System Process). (**C**) Scatter plots display the differential effects of each vitamin on the expression of immune-relevant genes. Each axis depicts the vitamin-induced log_2_ fold change as compared to the corresponding pathogen stimulation alone. Genes written in red indicate significant differences in their regulation induced by each vitamin (real *FC* > 3 between the fold changes induced by atRA and vitD during infections).

Next, we compared the effects exerted by both vitamins on the transcriptional response during infection. We could observe an important overlap in the regulatory potential of both vitamins. As shown in Fig. 8A, up to 444 genes of the vitD-regulated genes were also regulated by atRA. This fraction represents 71.2% of the vitD-regulated genes, but only 28.2% of the genes regulated by atRA. When we focused only on the immune-relevant genes, the distribution was similar. 217 genes were exclusively regulated by atRA and only 47 genes were vitD-specific. A total of 129 genes were regulated by both vitamins (Fig. 8B). When we analysed the regulation of these 129 genes in each of the pathogen settings, most of the genes were regulated in the same direction by both vitamins (Fig. 8C). Nevertheless, we could also observe differential modulatory effects by each of the vitamins as to the direction and extent of the regulation. Genes such as EDN1, BCL2 and CCL22 were up-regulated by vitD and down-regulated by atRA. Other genes such as CCL23 or CD300A were strongly up-regulated only by atRA in all settings. Differences in the magnitude of the regulation became apparent for genes such as CD14, which was strongly up-regulated by vitD during both fungal infections. In the *C. albicans* infection, we could observe significant differences in the magnitude of down-regulation of genes such as IL12A, CCL1 and IFNA14. A similar scenario is observed for *E. coli* stimulation, with a stronger impact of atRA on the differential expression of most genes, as shown for CSF2 and IL12B (Fig. 8C).

### Enrichment of RAREs and VDREs in DEGs

Both vitamin-specific nuclear receptors (RARs and VDRs) can form heterodimers with RXRs and bind similar response elements in regulatory regions of their target genes. We performed *in silico* analysis of putative binding sites for RARs and VDRs in the proximity of protein-coding genes, and examined the overlap of the identified binding sites with our transcriptional data. For atRA, we found a significant enrichment of RAREs in the regulatory regions of genes differentially expressed in response to atRA during both fungal infections (*p* = 0.0003 and *p* = 0.0029; Table 1). This enrichment could also be observed separately both for up- and for down-regulated genes. Interestingly, during *E. coli* infection the proportion of DEGs with RAREs was lower (23.2%) and did not reach significance level when compared to non-DEGs with response elements (Table 1).

On the other hand, for vitD-regulated genes, we could observe an enrichment in VDREs-containing genes among the up-regulated genes in response to vitD. The enrichment was significant in all three stimulatory settings. Nevertheless, we could not find VDRE enrichment among negatively regulated genes, with only seven of these genes presenting a VDRE in their regulatory regions (Table 2).

## Discussion

We used a high-throughput RNA-seq-based approach to characterize the whole immunomodulatory potential of the vitamins A and D during infections of bacterial and fungal origin. Using human monocytes as a host-cell model, we analysed the differential role of both vitamins upon four different stimulatory settings: upon *A. fumigatus* infection, upon *C. albicans* infection, upon *E. coli* infection or in absence of any inflammatory stimulus. Gene ontology and pathway analyses of the differential expression patterns were carried out to define the regulatory role of these vitamins upon each infection type, and to identify their underlying mechanisms.

We observed an important and specific impact of the inflammatory stimulus on the vitamin-mediated regulation of transcription. Especially in the case of vitamin D, where infection drastically reduced the amount of vitD-regulated genes when compared to its regulation in the absence of inflammatory stimulus (Fig. 2B). Also the relation of up- *vs.* down-regulated genes was shifted upon infection, with more up-regulated than down-regulated genes in response to vitD upon infection. Interestingly, the contrary could be observed for vitamin A, where atRA led to slightly more repression of gene expression than activation, only during infection (Fig. 2B). The surprising number of vitamin A-downregulated genes cannot be explained by the classical mechanisms of RAR-mediated regulation of transcription, with main focus on ligand-dependent activation of gene expression^1^. Moreover, also the recently identified non-genomic effects of atRA rely on the activation of signaling cascades^1^. Indeed, nuclear receptor-mediated repression and trans-repression pathways are largely unknown, and only few mechanisms have been described for glucocorticoid-receptors, peroxisome proliferator-activated receptor (PPAR)*γ* and liver-X receptors, but none for RARs^51^. Moreover, the question remained as to what extent certain groups of genes might be specifically up- or down-regulated by the vitamins during infection.

K-means clustering coupled with gene ontology enrichment analysis allowed us to identify different patterns of vitamin-mediated regulation depending on concomitant pathogen stimulation. Predominant enrichment of metabolic pathways was observed in gene clusters with none or minimal impact of pathogen stimulation, while the immunomodulatory role of vitamins was highlighted across pathogen-triggered clusters (Fig. 3). Overall, during inflammation, the GO category that was mostly enriched by vitamin stimulation was Immune System Process (GO:0002376). For vitamin D this enrichment could be confirmed for each of the infections. For atRA, Immune System Process ranked first during *C. albicans* and *E. coli* infection, but only 4th upon *A. fumigatus* infection (Fig. 4). This slight shift might be explained by a generally lower transcriptional response in response to *A. fumigatus*, with less immune-relevant genes to be susceptible to atRA-mediated regulation. Nevertheless, the huge importance of both vitamins as immunomodulators becomes even more evident considering the amount of immune-relevant genes (GO:0002376) regulated by atRA and vitD during infection: 346 and 176 genes, respectively. In addition, pathway analysis underpinned the notion that immune response is the most regulated biological function by both vitamins (Supplementary Table S2). Although the immunomodulatory effect of both vitamins has already been described for single genes in different cell models^20^,^21^,^23^,^24^,^52^,^53^,^54^, the dimension of this regulatory function has not been previously reported.

For all stimulations, we could describe an overwhelming and still unreported counteractive effect between the pathogen- and the vitamin-driven regulation of immune-relevant genes. Both vitamins, especially vitamin A, counteracted the transcriptional regulation in response to the pathogens (Fig. 5). The most prevalent expression dynamic was defined by genes that were up-regulated by the pathogens, and this effect reversed by vitamin A. The functional classification of the atRA-regulated genes allowed us to identify the cytokines as the best representatives of this dynamic. AtRA downregulated almost all cytokines, and a similar tendency was observed also for the chemokines. On the other hand, complement-activity genes were rather up-regulated by atRA. These findings might suggest a scenario in which atRA could lead to an attenuation of the immune response, in terms of pro-inflammatory cytokine release and immune cell recruitment, but sustain effective phagocytosis. This type of immunomodulation might have large-scale clinical potential, especially during systemic infections with hyper-inflammatory response, including *C. albicans* and *E. coli*-infections. During the last decade, much effort has been devoted to develop new therapeutic strategies for severe sepsis treatment, highlighting the need for new immunomodulatory agents^55^, especially since most of the proposed immunomodulators have failed to disclose any clinical benefit or have shown limited clinical efficacy^56^,^57^.

We could observe a high degree of specificity in the vitamin-mediated regulatory patterns between the different infection models. On the one hand, *E. coli* infection triggered the capability of vitamins to regulate gene expression, as compared to the other two infections. This might be attributed to the fact that *E. coli* stimulation led to the strongest transcriptional response, thereby presenting more targets susceptible for regulation by atRA or vitD. On the other hand, we could identify genes regulated by the vitamins in a pathogen-specific manner. Type-I interferons (IFN), for instance, were down-regulated by both vitamins exclusively upon *C. albicans*-infection. The type-I IFN response was identified in our dataset as a key signature of the *C. albicans*-induced inflammation, which is in agreement with previous reports^58^. Furthermore, Majer *et al*. could demonstrate that during systemic Candida infection type-I IFNs responses are associated with increased hyper-inflammation, tissue damage, and lethality^59^, highlighting the therapeutical need for immunomodulators able to repress this response.

In order to investigate possible indirect mechanisms leading to vitamin-mediated down-regulation of pro-inflammatory cytokines, we analysed the expression profiles of central regulators of the main signaling cascades. These included the Jak-STAT signaling, the NF*κ*B signaling or the MAPK signaling pathways, which upon vitamin stimulations showed significant enrichment in at least one of the stimulatory settings (data not shown). As shown by qPCR analysis, important activators of these pathways, such as BTK and PPP3CA were down-regulated by atRA after only three h of stimulation (Supplementary Fig. S4). Interestingly, both genes were also identified as potential keyplayers of the regulatory role of atRA in the consensus network inferred by the KeyPathwayMiner^43^,^44^ tool (Supplementary Fig. S3). We could also demonstrate the up-regulation of several inhibitory phosphatases by atRA. These included PTPN7, DUSP1 and DUSP7, which have shown to regulate the MAPK signaling pathway^60^,^61^. In addition, the ITIM-bearing inhibitory receptors CD300A and LILRB1 were already up-regulated by atRA after three hours of stimulation. Both receptors have been shown to modulate immune responses^62^,^63^. The transcriptional modulation of these genes might explain, at least in part, the regulatory effects of atRA observed after 6 h in our transcriptome data. Interestingly, none of them were differentially expressed upon vitamin D treatment, despite the reports pointing towards DUSP1 as central player in vitD-mediated regulation of immune functions^23^. An additional mechanism which could contribute to the immunomodulatory action of vitamins would be monocyte subpopulation differentiation. Nevertheless, despite a strong up-regulation of CD14, especially by vitamin D, we failed to find a particular pattern of subpopulation shift when analysing additional markers, such as CCR2 and CD16, at this early stage (data not shown). Further studies are needed to disclose the mechanisms of vitamin D-mediated repression of gene expression. Our data suggest the existence of indirect mechanisms, especially since we could not observe any enrichment of VDREs in down-regulated genes (Table 2), in contrast to the significant enrichment in up-regulated genes upon all types of infection. Interestingly, for atRA, we did observe significant enrichment of RAREs in genes that were down-regulated by the vitamin metabolite, at least during fungal infections. This might suggest the existence of direct RAR-mediated transrepression mechanisms. Upon *E. coli* challenge, we could not observe RARE enrichment in atRA-regulated genes (Table 1). It is likely that indirect and possibly non-genomic mechanisms^1^ are enabled upon *E. coli* infection, which might also explain the high amount of atRA-regulated genes during this type of infection. Further integrative analysis, correlating transcriptional data with ChIP-Seq analysis will help to clarify the mechanisms of RAR- and VDR-mediated repression of gene expression.

In conclusion, in the present study we have characterized, in a comprehensive manner, the immunomodulatory potential of vitamins A and D during infection. We observed that this potential is dependent on inflammatory stimulus and is to some extent specific for each of the pathogen scenarios tested. We described the undocumented ability of both vitamins to counteract the inflammatory response triggered by each pathogen. As a result, we observed a strong anti-inflammatory activity, especially by atRA, leading to the down-regulation of several cytokine- and chemokine-coding genes among others. Our study identified vitamins A and D as potent immunomodulators, that might be of particular importance in systemic infections, where the dysregulation of the immune response is responsible for the fatal outcome^55^,^59^,^64^. Moreover, recent studies have described important inadequacies of retinol^65^ and vitamin D^66^ in critically ill patients, and associated this deficiency with increased risk of mortality. Thus, monitoring the serum levels of vitamins A and D, as well as its adequate supplementation in individuals admitted in intensive care units, might have far-reaching prophylactic and therapeutic implications in severe infections.

## Additional Information

**Accession codes:** The raw Ion Proton sequence data in fastq format are stored in the Sequence Read Archive (SRA) at National Center for Biotechnology Information (NCBI) and can be accessed at NCBI homepage (accession number: SRP076532).

**How to cite this article:** Klassert, T. E. *et al*. Differential Effects of Vitamins A and D on the Transcriptional Landscape of Human Monocytes during Infection. *Sci. Rep.* **7**, 40599; doi: 10.1038/srep40599 (2017).

**Publisher's note:** Springer Nature remains neutral with regard to jurisdictional claims in published maps and institutional affiliations.

## Supplementary Material

Supplementary Information

Supplementary Dataset 1

Supplementary Dataset 2

## Figures and Tables

**Table 1:** Enrichment of RAREs in atRA-regulated genes during infection.

Comparison	Type of regulation	DEGs (FC > 2)	DEGs with RARE	Proportion of DEGs with RARE	Enrichment p-values	Significance level
**atRA on** ***A.f.*****-inf**	positive (UP)	302	79	0.261	**0.02239**	*
	negative (DOWN)	338	95	0.281	**0.002523**	**
	all	640	174	0.271	**0.0003018**	**
**atRA on** ***C.a.*****-inf**	positive (UP)	312	83	0.266	**0.03608**	*
	negative (DOWN)	326	88	0.269	**0.02413**	*
	all	638	171	0.268	**0.002911**	**
**atRA on** ***E.c.*****-inf**	positive (UP)	447	106	0.237	0.3113	ns
	negative (DOWN)	538	123	0.228	0.473	ns
	all	985	229	0.232	0.2465	ns

Enrichment of RAREs in the 10 kB-flanking regions of DEGs was calculated using Fisher’s exact test, comparing genes differentially regulated by atRA (*FC* > 2, *p* < 0.05) to those not differentially expressed. **p ≤ 0.01, *p ≤ 0.05.

**Table 2:** Enrichment of VDREs in vitD-regulated genes during infection.

Comparison	Type of regulation	DEGs (FC > 2)	DEGs with VDRE	Proportion of DEGs with VDRE	Enrichment p-values	Significance level
**vitD on** ***A.f.*****-inf**	positive (UP)	192	12	0.062	**0.01841**	*
	negative (DOWN)	106	5	0.047	0.2504	ns
	all	298	17	0.057	**0.01537**	*
**vitD on** ***C.a.*****-inf**	positive (UP)	169	13	0.076	**0.002324**	**
	negative (DOWN)	90	0	0	0.1772	ns
	all	259	13	0.05	0.05959	ns
**vitD on** ***E.c.*****-inf**	positive (UP)	224	14	0.062	**0.01604**	*
	negative (DOWN)	120	2	0.016	0.7758	ns
	all	344	16	0.046	0.1008	ns

Enrichment of VDREs in the 10 kB-flanking regions of DEGs was calculated using Fisher’s exact test, comparing genes differentially regulated by vitD (*FC* > 2, *p* < 0.05) to those not differentially expressed. **p ≤ 0.01, *p ≤ 0.05.
