# Supplementary material for: Differential Effects of Vitamins A and D on the Transcriptional Landscape of Human Monocytes during Infection
Source: Sci Rep. 2017 Jan 17;7:40599. doi: 10.1038/srep40599 (PMC5240108; doi:10.1038/srep40599)
Supplement: Supplementary Information [file srep40599-s1.pdf]

Supplementary information

Differential Effects of Vitamins A and D on the Transcriptional Landscape of Human Monocytes during Infection

Tilman E. Klassert, Julia Bräuer, Martin Hölzer, Magdalena Stock, Konstantin Riege, Cristina Zubiría-Barrera, Mario M. Müller, Silke Rummler, Christine Skerka, Manja Marz, and Hortense Slevogt

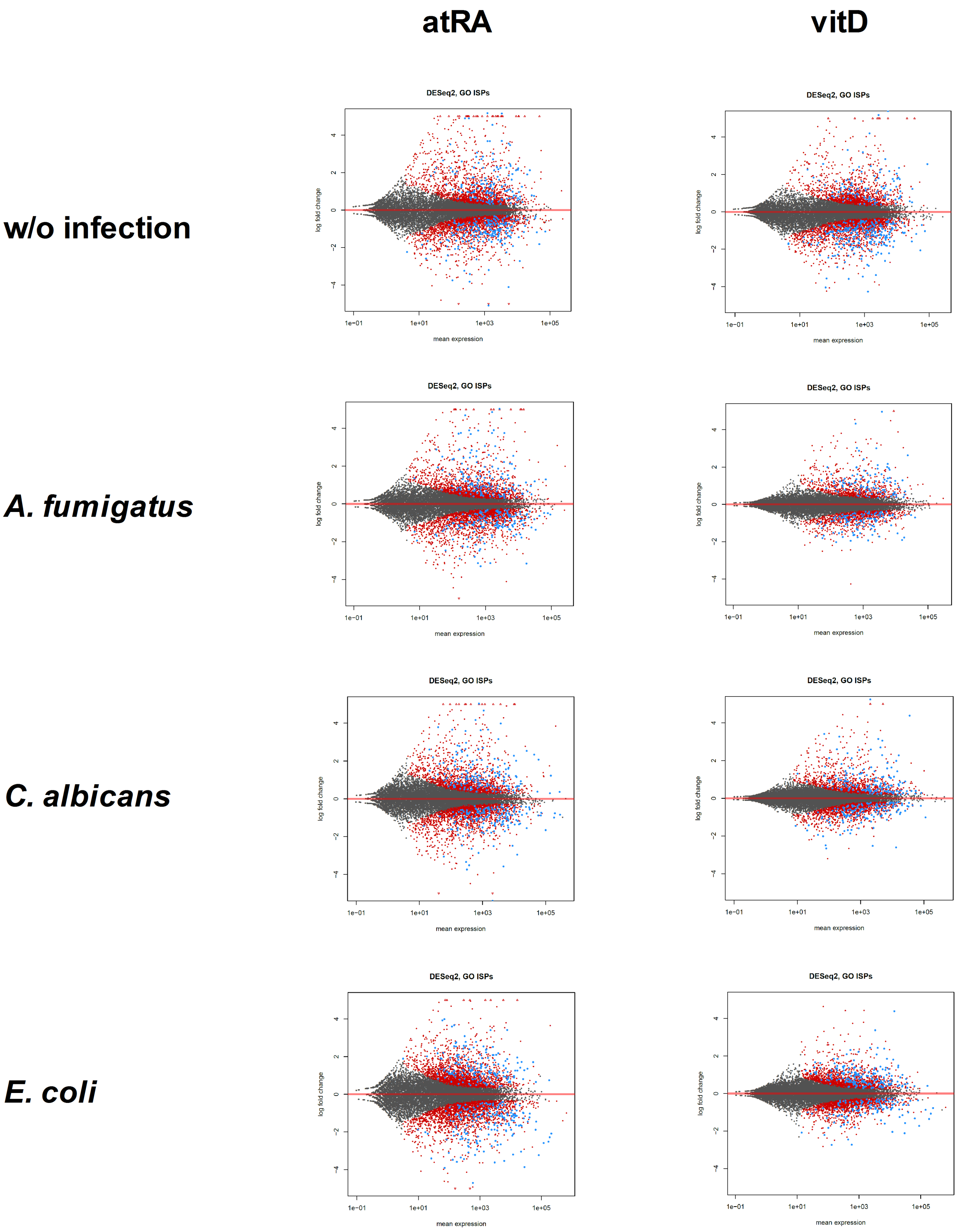

Supplementary Fig. S1 MA-plots: vitamin-dependent transcriptional profiles. Scatter plots displaying the mean expression and $\log_2$ fold changes (log2FC) of differentially expressed genes (DEGs) in response to atRA and vitD under each of the stimulatory settings (w/o infection, *A. fumigatus* infection, *C. albicans* infection, *E. coli* infection). Red dots represent significantly regulated genes. Blue dots represent DEGs belonging to GO category GO:0002376 (Immune System Process).

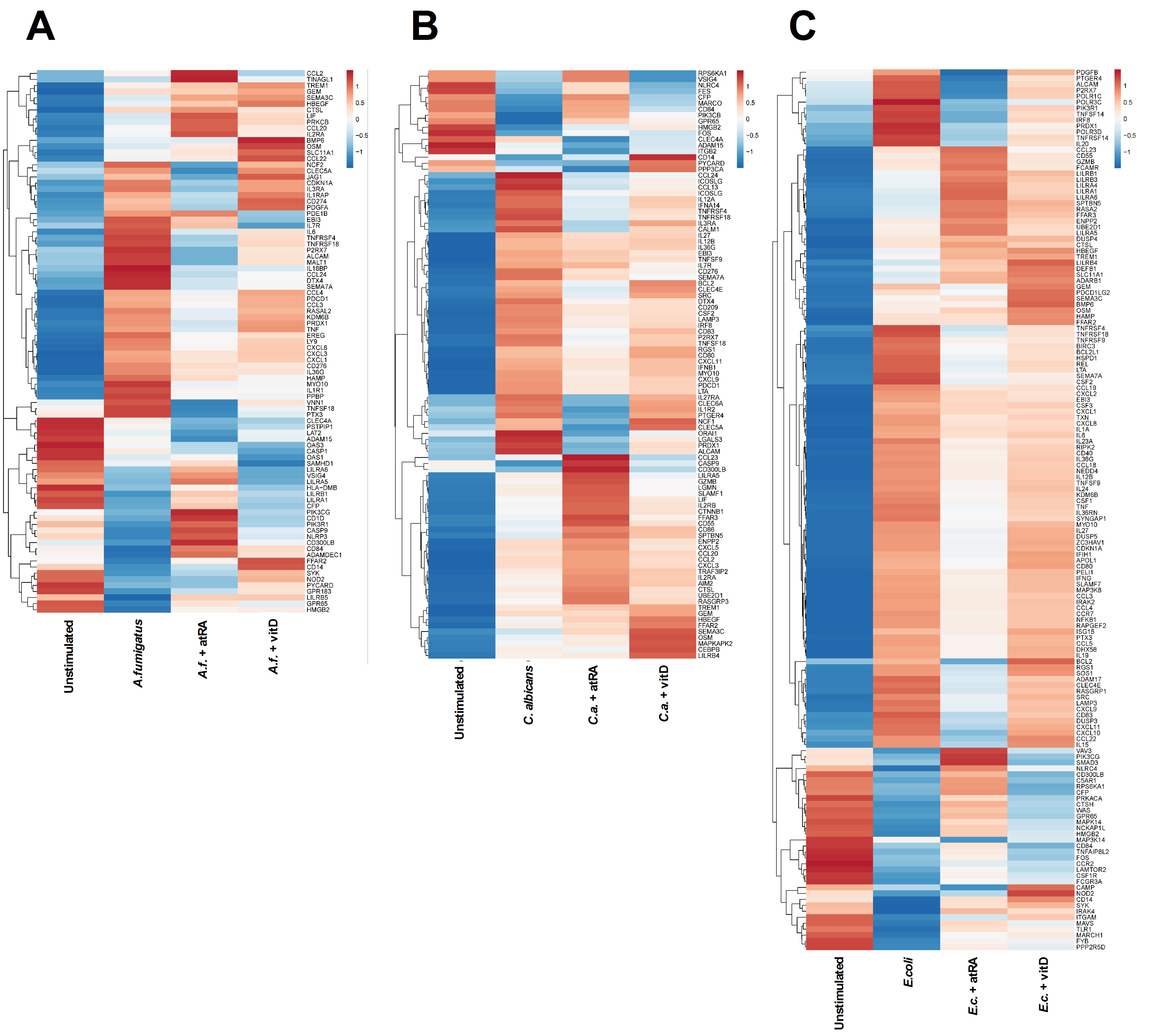

Supplementary Fig. S2 Hierarchical clustering of DEGs of GO:0006955 (Immune Response). Heat map of all genes differentially regulated (DEGs) by both the pathogens and any of the vitamins in each infection model. A: during infection with *A. fumigatus* and treatment with either vitamin A or D; B: during infection with *C. albicans* and treatment with either vitamin A or D; C: during infection with *E. coli* and treatment with either vitamin A or D.

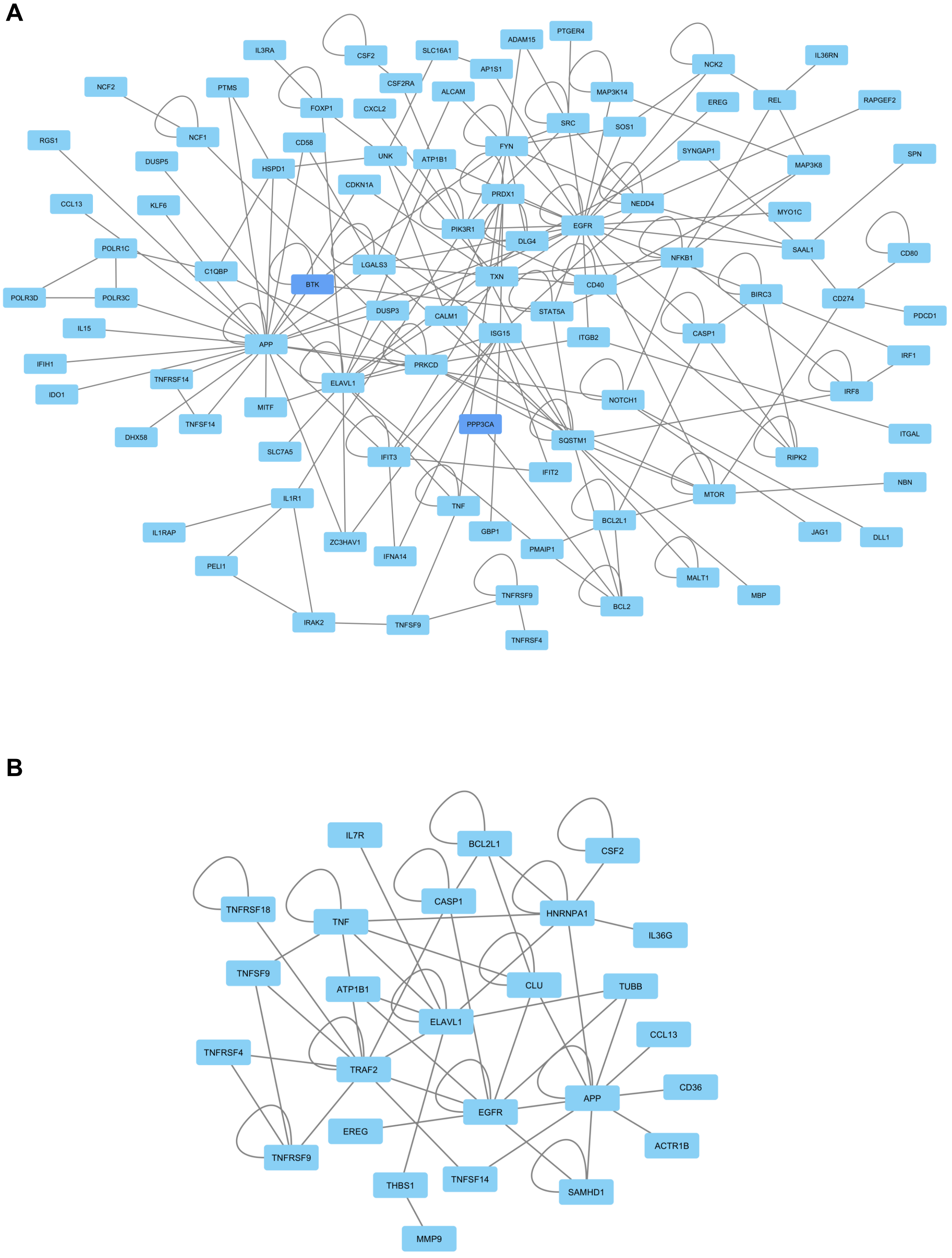

Supplementary Fig. S3 Consensus pathways of the immunomodulatory role of vitamins across all three infection types. After applying the `KeyPathwayMiner` tool[1,2], each network shows all genes that were down-regulated by atRA (A) or vitD (B) during all three infections as well as putative key regulators of their expression.

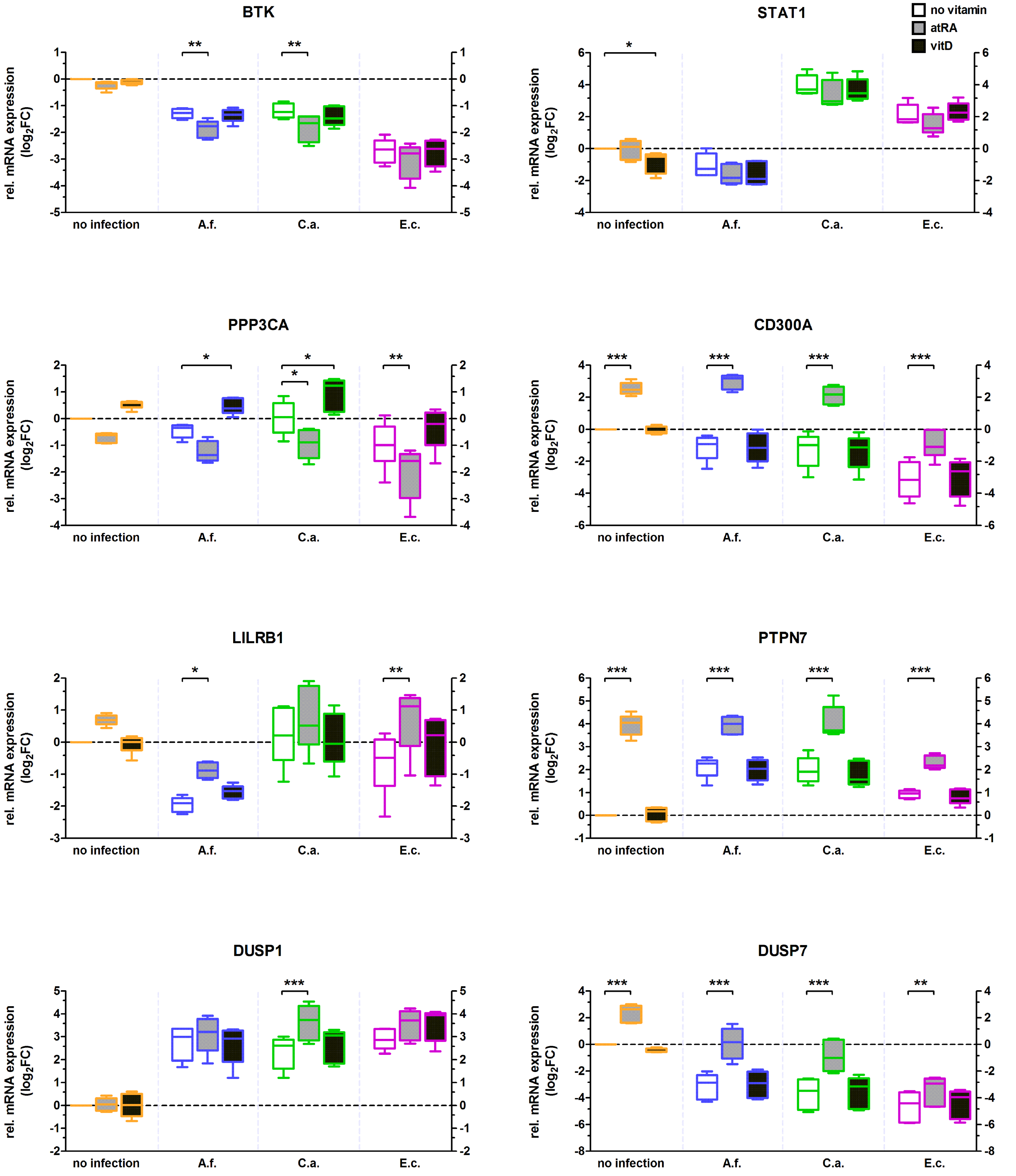

Supplementary Fig. S4 Analysis of the expression profiles of immunomodulatory genes in response to atRA and vitD after three hours of stimulation. Relative mRNA expression levels of selected genes measured by qPCR. Data were obtained from five independent experiments, each performed with cells from different donors. Statistical analysis was carried out by using repeated measures ANOVA and Bonferroni correction. Results are presented as mean *SEM* of the fold change relative to the control (unstimulated cells). *** $p \leq 0.001$, ** $p \leq 0.01$, * $p \leq 0.05$.

**Human gene**	**Symbol**	**Forward primer**	**Reverse primer**	**Size (bp)**
Bruton tyrosine kinase	*BTK*	TTGAAAAAGCCACTACCGCC	AATGTAGCCTTCCTGCCCAT	201
Cluster of differentiation 300a	*CD300A*	GACCATTCAGAGCTGTCCCA	GAATCAAACACCACCGAGGC	188
Dual specificity phosphatase 1	*DUSP1*	CCTTTCTGTACCTGGGCAGT	GCAGTGGACAAACACCCTTC	239
Dual specificity phosphatase 7	*DUSP7*	CTGCTCCTACAGAAGCTGCG	GAGCTGTCCACGTTGGTCT	113
Leukocyte immunoglobulin like receptor B1	*LILRB1*	TTCCCCATCCCATCCATCAC	TGAAGCCATCAAATGCCACC	217
Peptidylpropyl isomerase B	*PPIB*	ATGTAGGCCGGGTGATCTTT	TGAAGTTCTCATCGGGGAAG	219
Protein phosphatase 3 catalytic subunit alpha	*PPP3CA*	CCAAGGCAATTGATCCCAAG	CCAGCCTTCCCTCCTTCATAA	159
Protein tyrosine phosphatase, non-receptor type 7	*PTPN7*	AAAAACGCCAGCCAAGAAGC	CCCAGGGACCGAACGTC	84
Signal transducer and activator of transcription 1	*STAT1*	ACGCTGCCAATGATGTTTCA	ATCTCTGGGCGTTTTCCAGA	231

Supplementary Table S1 Overview of used qPCR-primers. Oligonucleotide sequences and expected amplicon sizes.

(A) atRA-regulated pathways

		atRA upon ***A. fumigatus*-infection**	atRA upon ***C. albicans*-infection**	atRA upon ***E. coli*-infection**	MEAN ENRICHMENT SCORE
**Cytokine-cytokine receptor interaction**	E. Score	4.365	9.147	17.53	10.347
	p-value	0.0127	0.0001	2.4E-08	
**Amoebiasis**	E. Score	12.958	8.954	7.813	9.908
	p-value	2.30E-06	0.0001	0.0004	
**Chemokine signaling pathway**	E. Score	4.706	3.423	12.13	6.753
	p-value	0.009	0.0322	5.40E-06	
**TNF-signaling pathway**	E. Score	6.579	4.819	7.987	6.462
	p-value	0.0014	0.0081	0.0003	
**Lysosome**	E. Score	3.612	5.371	9.019	6.001
	p-value	0.0269	0.0047	0.0001	
**Osteoclast differentiation**	E. Score	3.647	3.647	10.179	5.824
	p-value	0.0261	0.0261	3.80E-05	
**Rheumatoid arthritis**	E. Score	4.027	4.027	8.861	5.638
	p-value	0.0178	0.0178	0.0001	
**AGE-RAGE-signaling pathway**	E. Score	4.562	3.743	8.604	5.636
	p-value	0.0104	0.0237	0.0002	
**Hematopoietic cell lineage**	E. Score	6.45	6.45	3.343	5.414
	p-value	0.0016	0.0016	0.0353	
**Pathways in cancer**	E. Score	5.172	5.172	3.907	4.750
	p-value	0.0057	0.0057	0.0201	
**HIF-1 signaling pathway**	E. Score	3.906	3.139	4.597	3.881
	p-value	0.0201	0.0433	0.0101	

(B) vitD-regulated pathways

		vitD upon ***A. fumigatus*-infection**	vitD upon ***C. albicans*-infection**	vitD upon ***E. coli*-infection**	MEAN ENRICHMENT SCORE
**Cytokine-cytokine receptor interaction**	E. Score	5.495	9.595	18.508	11.199
	p-value	0.0041	6.81E-05	9.17E-09	
**Hematopoietic cell lineage**	E. Score	9.392	8.452	8.359	8.734
	p-value	8.33E-05	0.0002	0.0002	
**Amoebiasis**	E. Score	7.931	12.104	5.648	8.561
	p-value	0.0004	5.54E-06	0.0035	
**Pertussis**	E. Score	5.362	5.848	8.991	6.733
	p-value	0.0047	0.0029	0.0001	
**Tuberculosis**	E. Score	5.359	7.232	7.607	6.733
	p-value	0.0047	0.0007	0.0005	
**Legionellosis**	E. Score	5.394	5.827	7.873	6.364
	p-value	0.0045	0.0029	0.0004	
**TNF-signaling pathway**	E. Score	3.982	3.405	5.361	4.249
	p-value	0.0187	0.0332	0.0047	

Supplementary Table S2 Pathway analysis. Relation of all consensus pathways significantly ($p < 0.05$) enriched in all three stimulatory settings in response to either atRA (A) or vitD (B). Pathways are ordered by their mean enrichment score over all three settings.

References

1. Alcaraz, N. *et al.* Keypathwayminer 4.0: condition-specific pathway analysis by combining multiple omics studies and networks with cytoscape. *BMC Syst Biol* **8**, 99; DOI:10.1186/s12918-014-0099-x (2014).
2. Alcaraz, N. *et al.* Robust de novo pathway enrichment with keypathwayminer 5. *F1000Res* **5**, 1531; DOI:10.12688/f1000research.9054.1 (2016).
